# Evaluating evidence behind popular trauma narratives: neurobiological and treatment claims in *The Body Keeps the Score*

**DOI:** 10.1192/bjb.2025.10174

**Published:** 2026-08

**Authors:** Michael S. Scheeringa

**Affiliations:** Department of Psychiatry, Tulane University School of Medicine, New Orleans, Louisiana, USA

**Keywords:** Trauma and stressor-related disorders, causal inference, evidence-based mental health, neuroscience, psychological treatments

## Abstract

Bessel van der Kolk’s book *The Body Keeps the Score* has maintained exceptional cultural and clinical influence since its publication in 2014, remaining a best-seller and shaping public discourse on trauma. Its central claims – that trauma causes lasting neurobiological damage and that body-based treatments are uniquely effective – have been widely embraced but seldom subjected to systematic critical evaluation in peer-reviewed literature. This commentary synthesises the evidentiary basis for these claims as a counterweight to an influential narrative. It situates these findings within broader discussions of neuroscience framing, cultural appeal and evidence-based communication, underscoring the need for rigorous, balanced engagement with widely disseminated mental health narratives.

Over the past decade, popular science books have increasingly shaped public understanding of mental health, influencing clinical expectations, educational practices and even policy discussions. Among the most prominent is Bessel van der Kolk’s *The Body Keeps the Score*, first published in 2014, which has remained on best-seller lists for more than 8 years and continues to enjoy widespread readership among clinicians, educators and the general public. Its central thesis – that psychological trauma produces enduring changes in the brain and body, and that certain body-based treatments are uniquely effective – has become highly influential both within and beyond the mental health field.

Despite this sustained popularity and broad uptake, there has been remarkably little systematic critical evaluation of the empirical basis for the book’s claims in peer-reviewed literature. This is notable, given the book’s potential to shape treatment decisions, inform public policy and guide research priorities. Although many of its narratives and metaphors have clear appeal, the extent to which they accurately represent current scientific consensus remains an open question.

In a recent analysis, I examined 122 discrete claims from *The Body Keeps the Score*, including 42 on neurobiology, 51 on treatment efficacy and 29 on other topics, such as child development and memory.^1^ Van der Kolk – a pioneering researcher who conducted many of the early studies on post-traumatic stress disorder (PTSD) – supported many of his claims with research findings and voluminous citations. My review revealed multiple instances where cited evidence was incomplete, selectively presented, erroneously presented or inconsistent with broader research findings. The present commentary situates these findings within the broader scholarly and cultural context, considering how certain trauma narratives gain prominence, why neurobiological framings hold particular public appeal and what risks arise when complex research literatures are simplified for mass audiences.

## Neurobiology claims and the question of causality

One of the book’s most widely embraced ideas is that trauma inflicts direct, lasting damage on brain structures and neural networks involved in emotion regulation, memory and self-control. In addition, it is claimed to produce lasting damage to physical organs and systems, directly causing obesity, heart disease, autoimmune diseases and a wide range of other lethal conditions. Although neuroimaging studies have repeatedly documented structural and functional differences between individuals with and without PTSD, these were always cross-sectional designs – with no causal explanatory power – with findings that failed to replicate consistently.^2^ Further, these studies – extremely common in the peer-reviewed literature – rarely emphasise these ‘limitations’. When they are acknowledged, it is in cursory fashion with one or two sentences, while the central narratives portray confidence in trauma-inflicted damage.

There are three literature reviews of studies that measured neurobiological variables prior to trauma exposure and then followed the development of outcomes prospectively; all three concluded that differences likely precede trauma exposure.^3–5^ Even though prospective studies are more difficult to conduct than cross-sectional studies, the most recent of these reviews identified 25 such studies,^5^ and there have been half a dozen more since that publication. These reviews found little evidence for the book’s theory that trauma causes permanent brain damage. These findings are consistent with diathesis–stress models, which conceptualise such differences as vulnerability factors that increase the likelihood of developing PTSD following trauma.

The distinction between pre-existing vulnerabilities and trauma-induced damage is not a minor technicality – it has significant implications for how clinicians conceptualise resilience, prevention and treatment. Overemphasising damage models may unintentionally reinforce deterministic narratives, whereas recognising pre-existing vulnerabilities can encourage more targeted preventive strategies.

## Treatment claims and comparative efficacy

Another major theme in the book is the claim that ten ‘body-based’ treatments (psychomotor therapy, sensorimotor psychotherapy, somatic experiencing therapy, neurofeedback, eye movement desensitisation and reprocessing (EMDR) therapy, art, music, dance, theatre and yoga) are uniquely effective for trauma. It is claimed that they are the only types of treatment that can successfully process trauma and rewire brains, because trauma is embedded in the body, with many victims not even cognisant of experiencing trauma. These, and other body-based treatments, are a burgeoning industry of clinician workshops and retreats.^6^

Although a few of these approaches have shown promise in preliminary studies, the available evidence does not demonstrate superiority over established, evidence-based interventions such as cognitive–behavioural therapy (CBT). Meta-analyses and large randomised controlled trials consistently indicate that structured, trauma-focused talking therapies yield the most robust and durable outcomes.^7^ By contrast, studies of body-based interventions often face methodological limitations, including small sample sizes, lack of active control conditions and inconsistent follow-up data.^8^ EMDR is an exception among these treatments, being one of the two main recommended treatments for trauma, along with CBT, but it has not shown superiority to CBT. This does not mean such treatments lack value, but rather that their promotion as superior to well-validated approaches is not currently justified by the weight of evidence.

## Mechanisms of change and omitted evidence

If trauma damages the brain, identifying the mechanisms of such damage is essential. The book briefly mentions topics such as cortisol dysregulation and epigenetic changes, yet omits substantial discussion of the large – and largely inconclusive – literature on these topics. In the peer-reviewed literature, the early findings in stress-related cortisol research have proven difficult to replicate,^9^ and the field of trauma-related epigenetics is still characterised by small effect sizes, unclear mechanisms and failures to replicate.^10^

By contrast, alternative explanatory models, such as diathesis–stress frameworks, integrate neurobiological findings with well-established developmental and genetic influences on risk and resilience.^5^ These models offer a broader, evidence-consistent account of why trauma exposure results in persistent distress for some individuals but not for others.

## Why certain narratives prevail

The persistence and popularity of damage-focused neurobiological narratives cannot be understood solely in terms of selectively picked empirical support. Scholars have noted that neuroscience explanations often carry persuasive weight for both professionals and lay audiences – a phenomenon sometimes referred to as neurorealism, in which a theory possesses greater meaning because it is backed up by brain research. In the context of trauma, the idea that adversity leaves measurable ‘scars’ in the brain resonates with broader cultural movements towards validating lived experience, destigmatising mental illness and advocating for systemic change.

Although such aims are laudable, the uncritical acceptance of compelling but weakly supported narratives carries risks, including the potential to influence policy and clinical training in ways that are not supported by best available evidence. This underscores the need for continued scholarly engagement with widely disseminated mental health claims.

## Implications for clinical practice and public communication

Given the reach of *The Body Keeps the Score*, its claims are not merely academic – they shape patient expectations, influence practitioner training and inform public discourse on trauma. For clinicians, aligning practice with robust evidence is critical to ensuring that patients receive the most effective care. For researchers, this case highlights the importance of public scholarship that engages with popular mental health narratives while maintaining fidelity to empirical standards.

## Conclusion

The enduring popularity of trauma exposure as a superseding cause of an extraordinary array of diseases demonstrates the public’s deep interest in understanding trauma and recovery. However, the influence of such works heightens the responsibility of scholars and practitioners to critically evaluate the claims they advance. The evidence reviewed here suggests that key assertions in the book – particularly those concerning trauma-induced brain damage and the unique efficacy of body-based treatments – are not supported by the current weight of research. Moving forward, constructive engagement between researchers, clinicians and the public can help ensure that the compelling narratives driving cultural conversations about trauma are also anchored in scientific rigour.

## About the author

**Michael S. Scheeringa**, MD is an Adjunct Professor in the Department of Psychiatry at Tulane University School of Medicine, New Orleans, Louisiana, USA.
